# Febrile seizures: perceptions and knowledge of parents of affected and unaffected children

**DOI:** 10.1007/s00431-021-04335-1

**Published:** 2021-12-07

**Authors:** Steven Alan Rice, Ruth Melinda Müller, Sarah Jeschke, Birthe Herziger, Thilo Bertsche, Martina Patrizia Neininger, Astrid Bertsche

**Affiliations:** 1 University Hospital for Children and Adolescents, Universitätsmedizin Rostock, Ernst-Heydemann-Straße 8, Rostock, 18057 Germany; 2 Clinical Pharmacy, Institute of Pharmacy, Medical Faculty, Leipzig University and Drug Safety Center, Leipzig University and University Hospital, Bruederstraße 32, Leipzig, 04103 Germany

**Keywords:** Febrile seizures, Children, Perception, Fear, Antipyretics, Anti-seizure rescue medication

## Abstract

Febrile seizures (FS) in children are common, but little is known about parents’ perceptions and knowledge of FS. We interviewed parents of children aged 6 months to 6 years affected by FS (FS group, 65 parents) or unaffected (control group, 54 parents). In the FS group, 32% said they knew their child had an FS when the first event occurred, and 89% described fear when the child had a seizure, with a median intensity of 10/10 (Q25/Q75: 9/10). Related to follow-up, 77% in the FS group (will) observe their child more carefully after the first seizure happened, and 63% (will) give antipyretics earlier at a median temperature of 38.2 °C (100.8 °F). In the FS group, 62% were unaware of FS before the first event (54% of control group did not know about FS thus far, n.s.). In the FS group, 20% would put a solid object in the mouth of a child having a seizure (control group, 39%, *p* = 0.030), and 92% would administer an available anti-seizure rescue medication (control group, 78%, *p* = 0.019). In the FS group, 71% feared that children with FS might suffocate (control group, 70%, n.s.).

*Conclusion*: Information about FS and their management should be more available to improve parents’ coping and patient safety.

**Table:** 

**What is Known:** *• Febrile seizures in children are common.* *• The prognosis of children suffering from febrile seizures is usually rather good.*	
**What is New:** *• Over half of parents had not informed themselves about febrile seizures so far; and only 32% of parents realized their child had a febrile seizure when it occurred.* *• Most parents described own fear with a median intensity of 10/10; and 63% (will) give antipyretics earlier at a median temperature of 38.2 °C (100.8 °F).*

**Supplementary information:**

The online version contains supplementary material available at 10.1007/s00431-021-04335-1.

## Introduction

Febrile seizures in children are common, with an incidence of 2–5% in Europe and the USA [8, 10, 30]. They are defined as seizures occurring in children aged 6 months to 5 years with a body temperature > 38 °C (100.4 °F) without an intracranial cause, another definable cause of seizure such as electrolyte imbalance, or a history of an afebrile seizure [16]. The most common underlying conditions are respiratory tract infections and gastroenteritis [18]. A recent study showed that influenza and coronaviruses cause respiratory tract infections leading to a febrile seizure more often than other respiratory viruses [11]. According to the Generation R study, frequent fever episodes are associated with an increased risk of febrile seizures in the second and third years of life [32]. About one-third of children with a first febrile seizure have at least one recurrent febrile seizure [1]. A lower degree of fever in the initial febrile seizure is associated with an increased risk of subsequent seizures [6]. As febrile seizures can occur during a rise or drop in body temperature, they are at risk of being confused with afebrile seizures. Also, febrile seizures might be confused with self-limited seizures associated with episodes of gastroenteritis [28, 29].

Earlier studies reported insufficient knowledge and high amounts of anxiety in parents witnessing a febrile seizure in their child [2, 5, 7, 13, 14, 24, 26, 33, 34]. A questionnaire study from the Netherlands showed that parents’ anxiety about fever was associated with anxiety about recurrent febrile seizures [26]. In a study from Israel, all parents of children who experienced a febrile seizure reported anxiety [24]. A study from Switzerland showed that lack of knowledge about febrile seizures was associated with severe anxiety [7] as did a British study [2]. An investigation from Malaysia reported that many parents thought their child would die during a febrile seizure [5]. A Japanese study showed that parents without prior knowledge of febrile seizures managed the events less appropriately than parents who had prior knowledge [13]. The authors of a Swedish study concluded that parents were anxious during a febrile seizure because they did not adequately understand the event and did not know how to act in response to a febrile seizure [33]. A Turkish study reported that educational level and economic status influenced parents’ attitudes towards fever and febrile seizures [34]. A recent study from Germany confirmed that parents experiencing a febrile seizure expressed high levels of anxiety [14]. To address those problems, it is important to know if parents realize their child has a febrile seizure when the event occurs and how parents perceive the situation. To improve coping and patient safety, it is also necessary to be aware of the current parental knowledge of febrile seizures. This applies not only to parents of children affected by febrile seizures but also to parents with children in the age group at risk for febrile seizures but who have not yet been affected. As data on those aspects are scarce, we performed interviews to explore perceptions of parents whose children experienced a febrile seizure. Additionally, we wanted to learn about the knowledge of parents with children affected by febrile seizures in comparison to parents of unaffected children.

## Materials and methods

### Setting and patients

After approval by the local ethics committee, we performed this prospective observational study from October 2019 to March 2021 at a German university hospital for children and adolescents. We consecutively invited parents of children aged 6 months to 6 years who were admitted to our hospital due to at least one febrile seizure (FS group) to take part in the interview. We also invited parents of children who were admitted to our hospital for other diagnoses such as infections but had not experienced a febrile seizure thus far (control group). Patients diagnosed with epileptic disorder, malignant neoplasia, or meningitis were excluded from the study. As from our experience we expected approximately 60 patients with febrile seizures during the study period, we decided to also invite approximately 60 parents of children without febrile seizures. All interviews were conducted during the time of hospitalization. Parents were interviewed by a member of the study team on the ward during the daytime after at least 12 h in the hospital; all parents had already received information from the attending physician. Requirements to participate in the study were in-patient treatment, sufficient knowledge of the German language, and intellectual capacity to understand and answer the questions. If both parents were present, only one parent was interviewed. We let the parents decide who felt more comfortable answering the questions. Participation in this study was voluntary and without any compensation. Written informed consent was obtained from all participants.

### Data assessment

An expert panel, consisting of a neuropediatrician, pharmacists, a child and adolescent psychotherapist, and a medical student with a qualification as emergency medical technician, developed a questionnaire as basis for the structured interview. The questionnaire was pretested with 8 persons for comprehensibility, and the questions were adjusted accordingly. Answers to the open questions were clustered by the expert panel. First, categories were established by the expert panel after reviewing the original answers. The answers were then individually assigned to the categories by the panel members. In case of disagreement, a discussion and clarification within the panel was performed to reach consensus. In the interview, we addressed the following questions:

Parents’ perceptions of febrile seizures in the FS group:

1. Did you realize your child had a febrile seizure? If no, what did you think happened?
2. What was the dominating feeling when your child had a febrile seizure?
3. Who told you your child had a febrile seizure (pre-set answers to choose)?
4. Which issues were addressed in the first consultation about the diagnosis (pre-set answers to choose)?
5. Changes after the febrile seizure (for parents of children with a previous febrile seizure/for parents with a first febrile seizure): Were there/will there be any general changes in the handling of your child? Do you/will you take your child’s temperature more often? Do you/will you give your child medicine to lower his/her fever earlier? At what temperature do you/will you start giving your child medicine to lower his/her fever? Do you/will you see your pediatrician more often when your child has a fever?
  Parents’ knowledge of febrile seizures in the FS group and in the control group:
6. For parents in the FS group: Did you inform yourself on the topic of febrile seizures before your child was affected? For parents in the control group: Have you informed yourself on the topic of febrile seizures yet?
7. Which measures would you take in case of a febrile seizure (pre-set answers to choose)?
8. Do you know about possible negative consequences resulting from a febrile seizure (pre-set answers to choose)?
9. Do you feel adequately prepared for a febrile seizure in your child? What measures would you need to feel better prepared?

At the end of the interview, we asked the parents about sociodemographic data.

For more detailed information on the questionnaire, please see supplement 1.

### Statistics

Frequencies are reported in absolute and relative numbers. Continuous data are described as median with first (25%) and third (75%) quartile (Q25/Q75). To compare groups, we performed Mann–Whitney *U* tests for continuous data, and chi-square or Fisher’s exact tests as appropriate for nominal data. A *p* value ≤ 0.05 was considered to indicate significance.

## Results

**Fig. 1:**
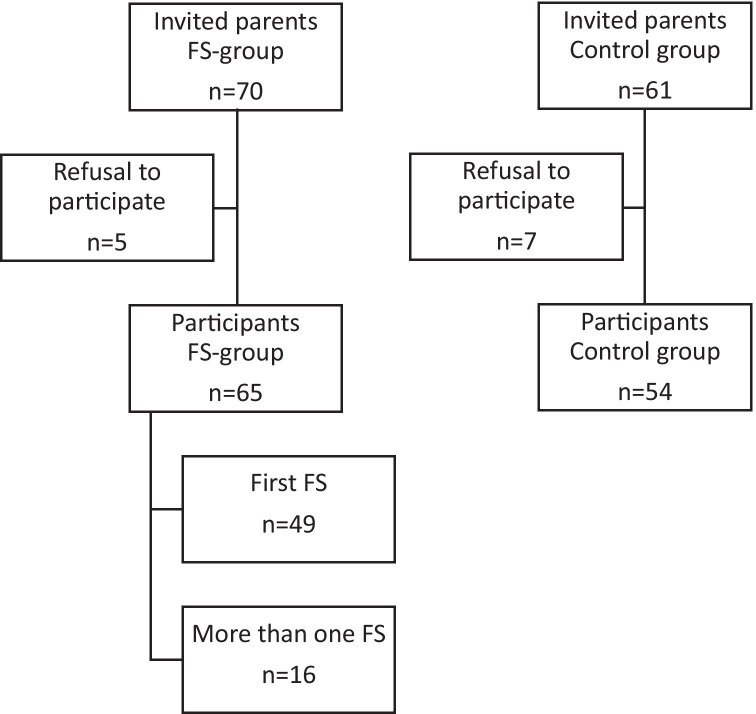
Flow chart showing patient enrollment

Altogether, 119 parents took part in the interview, 65 whose child had at least one febrile seizure (FS group) and 54 whose child had not (control group). Details on patient enrollment are shown in Fig. 1; sociodemographic data are displayed in Table 1.

**Table 1:** Parents’ and patients’ characteristics of children affected (FS-group) and unaffected by febrile seizures (control group)

Parameter	FS group	Control group	Significance
Parents (*n*)	65	54	
Female [*n* (%)]	49 (75)	42 (78)	n.s
Median age (Q25/Q75; min/max) [years]	34.0 (31.0/37.0; 21.0/45.0)	34.0 (29.8/36.3; 22.0/44.0)	n.s
Education of the parents			n.s
No school graduation	0 (0)	1 (2)	
“Hauptschule”/”Realschule” (schools that prepare students mainly for vocational careers) [*n* (%)]	44 (68)	26 (48)	
“Abitur” (i.e., the diploma for entry into higher education) [*n* (%)]	20 (31)	27 (50)	
No statement [*n* (%)]	1 (2)	0 (0)	
Profession of the parents			n.s
Medical [*n* (%)]	14 (22)	8 (15)	
Other [*n* (%)]	49 (75)	43 (80)	
No statement [*n* (%)]	2 (3)	3 (6)	
Hospitalized children (n)	65	54	
Female [*n* (%)]	26 (40)	25 (46)	n.s
Median age (Q25/Q75; min/max) [months]	21 (13/30.5; 8/65)	18 (13/33.8; 8/66)	n.s
Current febrile seizure [*n* (%)]			
Simple febrile seizure	51 (78)		
Complex febrile seizure	14 (22)		
Number of febrile seizures [*n* (%)]			
First seizure	49 (75)	-	
Second seizure	10 (15)	-	
More than two seizures	6 (9)	-	
Number of children with fever associated with the current hospitalization [*n* (%)]	65 (100)	29 (54)	

### Parents’ perceptions of febrile seizures in the FS group

In the FS group, 21/65 (32%) said when the first event occurred, they knew the child was having a febrile seizure; 41/65 (63%) had different ideas, for example, an epileptic seizure (11/65, 17%) or a life-threatening event (13/65, 20%), but did not realize the specific diagnosis. Three of the parents (3/65, 5%) did not feel  able to provide an answer to the question.

Asked about the feelings the first event induced in them, 58/65 (89%) described fear, 4/65 (6%) said they felt despair, 1 (2%) parent said he/she stayed calm, 1 (2%) did not witness the seizure, and 1 (2%) did not answer the question. On a scale from 0 to 10 (highest intensity), the participants who had reported fear described a median intensity of fear of 10 (Q25/Q75: 9/10). Those participants who described despair said the intensity of this feeling was in median 10 (Q25/Q75: 9.5/10).

According to the parents, 56/65 (86%) were informed by a physician about the diagnosis, 1/65 (2%) said the information was given by a medical assistant, 1/65 (2%) referred to his/her own professional medical experience, 6/65 (9%) claimed no one had informed them, and 1/65 (2%) did not remember who gave the information.

Concerning the consultation after the febrile seizure, 41/65 (63%) parents said they received information about acute measures to be taken in case of another febrile seizure. More details on the topics parents claimed they were informed about are displayed in Table 2.

**Table 2:** Share of parents of children affected by one febrile seizure (*n* = 49) and by more than one febrile seizure (*n* = 16) who claimed the respective topic was addressed in the physician’s consultation about febrile seizures. The number of parents who did not feel comfortable providing a statement is presented in curly brackets

Topic	Parents of children with one febrile seizure *n* (%)	Parents of children with more than one febrile seizure *n* (%)	Significance
Acute measures to be taken in case of a febrile seizure	29 (59%) {6 (12%)}	12 (75%) {1 (6%)}	n.s
Long-term consequences of febrile seizures	24 (49%) {5 (10%)}	7 (44%) {3 (19%)}	n.s
Frequency of febrile seizures	24 (49%) {6 (12%)}	6 (38%) {3 (19%)}	n.s
Measures to prevent febrile seizures	19 (39%) {5 (10%)}	10 (63%) {1 (6%)}	n.s
Risk factors for febrile seizures	22 (45%) {5 (10%)}	6 (38%) {2 (13%)}	n.s
Acute complications of febrile seizures	22 (45%) {7 (14%)}	4 (25%) {2 (13%)}	n.s

Fifty of the parents (50/65, 77%) said that after the febrile seizure, there had been/would be general changes in the handling of their child such as closer observation. As a specific change, 41/65 (63%) give (will give) antipyretics earlier at a median temperature of 38.2 °C (100.8 °F; Q25/Q75: 38.0 °C/38.5 °C [100.4 °F/101.3 °F]). For more details on changes, see Table 3.

**Table 3:** Changes that occur(red) after the febrile seizure of their child according to the parents of children affected by one febrile seizure (*n* = 49) or more than one febrile seizure (*n* = 16). Parents of children with one febrile seizure were asked about their future practice; parents of children with more than one febrile seizure were interviewed about their current practice. The number of parents who did not feel comfortable providing a statement is presented in curly brackets

Question	Parents of children with one febrile seizure n (%)	Parents of children with more than one febrile seizure *n* (%)	Significance
Were there/will there be any general changes in the handling of your child? [*n* (%) of parents answering “yes”]	37 (76%) {0 (0%)}	13 (81%) {0 (0%)}	n.s
Do you/will you take your child’s temperature more often? [*n* (%) of parents answering “yes”]	34 (69%) {2 (4%)}	12 (75%) {0 (0%)}	n.s
Do you/will you give your child medicine to lower his/her fever earlier? [*n* (%) of parents answering “yes”]	28 (57%) {1 (2%)}	13 (81%) {0 (0%)}	n.s
At what temperature do you/will you start giving your child medicine to lower his/her fever? [median (Q25/Q75)]	38.3 °C (38.0 °C/38.5 °C) 100.9 °F (100.4 °F/101.3 °F) {21 (43%)}	38.0 (37.9 °C/38.5 °C) 100.4 °F (100.2 °F/101.3 °F) {5 (31%)}	n.s
Do you/will you see your pediatrician more often when your child has a fever? [*n* (%) of parents answering “yes”]	13 (27%) {3 (6%)}	3 (19%) {1 (6%)}	n.s

### Parents’ knowledge of febrile seizures in the FS group and in the control group

In the FS group, 40/65 (62%) had not informed themselves about febrile seizures before the first event happened. In the control group, 29/54 (54%) had not yet informed themselves about febrile seizures (FS group vs control group: n.s.). Asked about measures they would take in case of a febrile seizure, 13/65 (20%) of the FS group would put a solid object in the mouth of a child having a seizure (control group, 21/54 (39%), *p* = 0.030); 60/65 (92%) in the FS group would administer an available rescue medication (control group, 42/54 (78%), *p* = 0.019). More information about measures parents would take in case of a febrile seizure can be found in Table 4. On the question relating to possible negative consequences of febrile seizures, 46/65 (71%) of the FS group stated children can suffocate (control group, 38/54 (70%), n.s.), 27/65 (42%) stated children with febrile seizures can develop epilepsy (control group, 26/54 (48%), n.s.), and 27/65 (42%) stated it can lead to a developmental disorder (control group, 25/54 (46%), n.s.). Detailed information about assumed consequences of a febrile seizure is displayed in Table 5. On a scale from 1 (not at all) to 5 (definitely), the FS group described their preparedness for another febrile seizure in median as 4 (Q25/Q75: 2/4); the parents of the control group rated their preparedness for a febrile seizure as 2 (Q25/Q75: 1/3), *p* < 0.001. In the FS group, 2 parents did not feel able to judge their preparedness. Asked in an open question what would be necessary to be better prepared for a febrile seizure, the following answers were given most often: general information on febrile seizures by pediatricians/medical staff before the first event (FS group, 10/65 (13%), control group, 40/54 (74%), *p* < 0.001), training on measures to be taken in case of a febrile seizure/pediatric emergency by pediatricians or medical staff (FS group, 8/65 (10%), control group, 24/54 (44%), *p* < 0.001), information on febrile seizures/pediatric emergencies in doctor’s offices (FS group, 5/65 (6%), control group, 25/54 (46%), *p* < 0.001), information about febrile seizures in the internet (FS group, 1 (2%), control group, 5 (9%), n.s.). In the FS group, 9/65 (14%) said such a situation will always be overwhelming and parents will never be properly prepared (control group, 1 (2%), *p* = 0.021).

**Table 4:** Share of parents who would take the respective measure in case of a febrile seizure of their child. The number of parents who did not feel able comfortable providing a statement is presented in curly brackets

	FS group (*n* = 65)	Control group (*n* = 54)	Significance
Clear the surroundings	61 (94%) {0 (0%)}	44 (81%) {0 (0%)}	0.037
Calm the child	60 (92%) {0 (0%)}	48 (89%) {0 (0%)}	n.s
Administer an anti-seizure rescue medication if available	60 (92%) {1 (2%)}	42 (78%) {1 (2%)}	0.019
Call the emergency	59 (91%) {1 (2%)}	51 (94%) {0 (0%)}	n.s
Document the length of the seizure	50 (77%) {2 (3%)}	21 (39%) {1 (2%)}	< 0.001
Administer fever medication	29 (45%) {4 (6%)}	13 (24%) {0 (0%)}	0.009
Put a solid object into the child’s mouth	13 (20%) {2 (3%)}	21 (39%) {0 (0%)}	0.030

**Table 5:** Share of parents who thought the respective consequences could result from a febrile seizure. The number of parents who did not feel comfortable providing a statement is presented in curly brackets

	FS group (*n* = 65)	Control group (*n* = 54)	Significance
Injuries	60 (92%) {0 (0%)}	51 (94%) {0 (0%)}	n.s
Other febrile seizures	58 (89%) {2 (3%)}	51 (94%) {0 (0%)}	n.s
Suffocation of the child	46 (71%) {3 (5%)}	38 (70%) {4 (7%)}	n.s
Epilepsy	27 (42%) {8 (12%)}	26 (48%) {3 (6%)}	n.s
Developmental disorder	27 (42%) {1 (2%)}	25 (46%) {0 (0%)}	n.s

## Discussion

### Parents’ perceptions of febrile seizures in the FS group

Only one-third of the parents of affected children said they knew the child experienced a febrile seizure when the event occurred, and most parents in our study expressed a maximum level of anxiety when experiencing their child’s febrile seizure. This is in accordance with other studies that also report high levels of anxiety in parents experiencing a febrile seizure of their child [2, 5, 7, 14, 24, 26, 33, 34]. Lack of parents’ knowledge about febrile seizures was also shown in earlier studies and might enhance anxiety and hinder parents from undertaking appropriate actions to manage the seizure [13]. A recent consensus statement on the information that should be delivered when a child has a febrile seizure recommended addressing definition, parental stress, the commonness of febrile seizures, measures to be taken when a seizure occurs, and prognosis [17]. Asked what specific information they received, two-thirds of affected parents in our study said they received information about measures to be taken if their child had another febrile seizure. Fewer than half of the participants said they were given information on topics such as frequency of or risk factors for febrile seizures, acute complications, measures to prevent seizures, and long-term consequences. We were not able to judge if this information was really not given or if the parents were so emotionally overwhelmed that they were not able to process or absorb the information provided. Thus, information should be given in a structured way and be repeated regularly. Furthermore, oral information should be supported by written leaflets [21].

In accordance with other studies [33], parents in our study who had experienced a febrile seizure of their child tend to watch their children very carefully; in particular, they take their children’s temperature more often and use antipyretics very early at a median temperature of 38.2 °C (100.8 °F). It is important to tell parents that prophylactic fever medication at low temperatures in most cases cannot prevent febrile seizures. Otherwise, they might feel guilty when a febrile seizure occurs. A systematic review published in 2013 found that antipyretics were ineffective in reducing the recurrence rate of febrile seizures [23]. A recent review showed only weak evidence for a possible role of antipyretics for preventing a febrile seizure in the same fever episode and stated that febrile seizures in future fever episodes clearly cannot be prevented [9].

### Parents’ knowledge of febrile seizures in the FS group and in the control group

Many participants said they had not informed themselves about febrile seizures when they were not or not yet affected. In addition to enhancing parents’ anxiety, this can lead to reduced patient safety. For example, the number who would perform the obsolete action of putting a solid object in their child’s mouth during a seizure was twice as high in parents whose children had not had a febrile seizure compared to those whose children had. As it is not predictable if a child will experience a febrile seizure, all parents should be informed about this possible emergency situation in order to act appropriately. A high share of parents in both groups expressed willingness to administer an available anti-seizure rescue medication. The parents should be told that most febrile seizures do not last longer than 2 to 3 min and do not require any treatment [17]. They should, however, be encouraged to administer an anti-seizure rescue medication for seizures lasting longer than 5 min, to reduce the risk of a prolonged seizure and its complications [12, 31].

Asked for possible consequences of febrile seizures, more than 40% of participants said there is a risk for epilepsy and for developmental disorder. Studies conducted in the 1960s and 1970s found a 3% and 2% epilepsy risk for children with febrile seizures [25] [19]. A review from 2008 showed a slightly higher risk between 2.0% and 7.5% of later epilepsy after a febrile seizure [4]. Consequently, the general prognosis is still favorable as more than 90% of children suffering from a febrile seizure do not develop epilepsy. With regard to intellectual and behavioral outcomes of children with febrile seizures, a population-based study of children born in one week in April 1970 in the UK found no differences between children with and without febrile seizures [27]. A recent study from Sweden, however, showed that children with early onset of febrile seizures and those with recurrent febrile seizures may be at risk for developing cognitive executive dysfunctions [3]. Nevertheless, the developmental prognosis is usually considered to be relatively favorable. As we did not ask for the grade of probability of development of an epilepsy or a developmental disorder, we were not able to judge if the parents interviewed in our study were aware of the slightly increased risk or if they overestimated those risks.

Parents who had not experienced a febrile seizure in their child so far felt badly prepared for such an event. Accordingly, they expressed the wish to receive information from their healthcare providers. A previous study had already shown the importance of healthcare professionals’ support to handle the situation [33]. This should include provision of adequate contact details of medical services [20]. It should be kept in mind that it is important not only to give the parents adequate information and guidance on management of childhood fever and febrile seizures [15, 20]. Healthcare professionals should also support parents emotionally to enable them to better cope with the situation of a febrile seizure. This includes also addressing the parents’ anxiety [22] and speaking to parents about the usually benign nature of the phenomenon [20].

## Limitations

The study was performed in a single center. The questionnaire was not validated.

As parents had already received information from the treating physician prior to the interview, this could lead to parents' responses mainly reflecting the information they had just received from the treating physician, his or her ability to communicate and the completeness of the information he or she gave, more than the parents' prior or actual knowledge. Moreover, some parents could have answered differently if they had been interviewed immediately after discharge, i.e., after possibly receiving some more information by the attending physician. Some of the answers might not fully reflect the actual and future practice and might be influenced by the recent episode of a febrile seizure in the affected group or the cause for admission in the control group. We did not ask the parents to assume the extent of risk increase for the development of epilepsy or a developmental disorder. Thus, we were not able to judge if the interviewed parents were aware of the slightly increased risks or if they simply overestimated them.

## Conclusion

Only one-third of parents realized their child had a febrile seizure when the event occurred. Most parents of children with febrile seizures expressed extremely high levels of anxiety. The knowledge of parents of both affected and unaffected children is insufficient. In order to facilitate parents’ coping and to improve patient safety, parents should receive more information on this topic and more emotional support. Information could easily be distributed by pediatricians’ offices.

## Supplementary information

Below is the link to the electronic supplementary material.

Supplementary file1 (PDF 123 KB)

## Data Availability

The data presented in this study are available on request from the corresponding author.

## Abbreviation

FS: Febrile seizures

## References

[1] Berg AT, Shinnar S, Darefsky AS, Holford TR, Shapiro ED, Salomon ME, Crain EF, Hauser AW (1997). Predictors of recurrent febrile seizures. a prospective cohort study. Arch Pediatr Adolesc Med.

[2] Besag FM, Nomayo A, Pool F (2005). The reactions of parents who think that a child is dying in a seizure–in their own words. Epilepsy Behav.

[3] Billstedt E, Nilsson G, Leffler L, Carlsson L, Olsson I, Fernell E, Gillberg C (2020). Cognitive functioning in a representative cohort of preschool children with febrile seizures. Acta Paediatr.

[4] Chungath M, Shorvon S (2008). The mortality and morbidity of febrile seizures. Nat Clin Pract Neurol.

[5] Deng CT, Zulkifli HI, Azizi BH (1996). Parental reactions to febrile seizures in Malaysian children. Med J Malaysia.

[6] El-Radhi AS (1998) Lower degree of fever at the initial febrile convulsion is associated with increased risk of subsequent convulsions. Eur J Paediatr Neurol 2:91–96. 10.1016/s1090-3798(98)80047-0 10.1016/s1090-3798(98)80047-0 10724102

[7] Flury T, Aebi C, Donati F (2001). Febrile seizures and parental anxiety: does information help?. Swiss Med Wkly.

[8] Forsgren L, Sidenvall R, Blomquist HK, Heijbel J (1990) A prospective incidence study of febrile convulsions. Acta Paediatr Scand 79:550–557. 10.1111/j.1651-2227.1990.tb11510.x 10.1111/j.1651-2227.1990.tb11510.x 2386045

[9] Hashimoto R, Suto M, Tsuji M, Sasaki H, Takehara K, Ishiguro A, Kubota M (2021). Use of antipyretics for preventing febrile seizure recurrence in children: a systematic review and meta-analysis. Eur J Pediatr.

[10] Hauser WA (1994). The prevalence and incidence of convulsive disorders in children. Epilepsia.

[11] Hautala M, Arvila J, Pokka T, Mikkonen K, Koskela U, Helander H, Glumoff V, Rantala H, Tapiainen T (2021). Respiratory viruses and febrile response in children with febrile seizures: a cohort study and embedded case-control study. Seizure.

[12] Hesdorffer DC, Shinnar S, Lax DN, Pellock JM, Nordli DR, Seinfeld S, Gallentine W, Frank LM, Lewis DV, Shinnar RC, Bello JA, Chan S, Epstein LG, Moshé SL, Liu B, Sun S, FEBSTAT study team,  (2016). Risk factors for subsequent febrile seizures in the FEBSTAT study. Epilepsia.

[13] Kanemura H, Sano F, Mizorogi S, Tando T, Sugita K, Aihara M (2013). Parental thoughts and actions regarding their child’s first febrile seizure. Pediatr Int.

[14] Klotz KA, Özcan J, Sag Y, Schönberger J, Kaier K, Jacobs J (2021). Anxiety of families after first unprovoked or first febrile seizure - a prospective, randomized pilot study. Epilepsy Behav.

[15] Laino D, Mencaroni E, Esposito S (2018) Management of pediatric febrile seizures. Int J Environ Res Public Health 15:2232. 10.3390/ijerph15102232 10.3390/ijerph15102232 PMC6210946 30321985

[16] Leung AK, Hon KL, Leung TN (2018). Febrile seizures: an overview. Drugs. Context.

[17] Loussouarn A, Devlin A, Bast T, Benoist G, Corrard F, Cross H, Ferretti A, Viguer FG, Guerrini R, Klepper J, Meissner T, Milh M, Poltorak V, Raucci U, San Antonio-Arce V, Sie A, Smeyers P, Specchio N, Sutcliffe A, Trauffler A, Dozières-Puyravel B, Auvin S (2021). Consensus statements on the information to deliver after a febrile seizure. Eur J Pediatr.

[18] Mahdi AH, Taha SA (1982). The first febrile convulsion: an analysis of 108 children in Saudi Arabia. Ann Trop Paediatr.

[19] Nelson KB, Ellenberg JH (1978). Prognosis in children with febrile seizures. Pediatrics.

[20] Offringa M, Newton R, Cozijnsen MA, Nevitt SJ (2017) Prophylactic drug management for febrile seizures in children. Cochrane Database Syst Rev 2:CD003031. 10.1002/14651858.CD003031.pub3 10.1002/14651858.CD003031.pub3 PMC6464693 28225210

[21] Paul F, Jones MC, Hendry C, Adair PM (2007). The quality of written information for parents regarding the management of a febrile convulsion: a randomized controlled trial. J Clin Nurs.

[22] Paul SP, Rogers E, Wilkinson R, Paul B (2015). Management of febrile convulsion in children. Emerg Nurse.

[23] Rosenbloom E, Finkelstein Y, Adams-Webber T, Kozer E (2013). Do antipyretics prevent the recurrence of febrile seizures in children? A systematic review of randomized controlled trials and meta-analysis. Eur J Paediatr Neurol.

[24] Shuper A, Gabbay U, Mimouni M (1996). Parental anxiety in febrile convulsions. Isr J Med Sci.

[25] Van der Berg BJ, Yerushalmy J (1969). Studies on convulsive disorders in young children. I. Incidence of febrile and nonfebrile convulsions by age and other factors. Pediatr Res.

[26] Van Stuijvenberg M, de Vos S, Tjiang GC, Steyerberg EW, Derksen-Lubsen G, Moll HA (2007). Parents' fear regarding fever and febrile seizures. Acta Paediatr.

[27] Verity CM, Greenwood R, Golding J (1998). Long-term intellectual and behavioral outcomes of children with febrile convulsions. N Engl J Med.

[28] Verrotti A, Moavero R, Vigevano F, Cantonetti L, Guerra A, Spezia E, Tricarico A, Nanni G, Agostinelli S, Chiarelli F, Parisi P, Capovilla G, Beccaria F, Spalice A, Coppola G, Franzoni E, Gentile V, Casellato S, Veggiotti P, Malgesini S, Crichiutti G, Balestri P, Grosso S, Zamponi N, Incorpora G, Savasta S, Costa P, Pruna D, Cusmai R (2014). Long-term follow-up in children with benign convulsions associated with gastroenteritis. Eur J Paediatr Neurol.

[29] Verrotti A, Nanni G, Agostinelli S, Parisi P, Capovilla G, Beccaria F, Iannetti P, Spalice A, Coppola G, Franzoni E, Gentile V, Casellato S, Veggiotti P, Malgesini S, Crichiutti G, Balestri P, Grosso S, Zamponi N, Incorpora G, Savasta S, Costa P, Pruna D, Chiarelli F (2011). Benign convulsions associated with mild gastroenteritis: a multicenter clinical study. Epilepsy Res.

[30] Vestergaard M, Obel C, Henriksen TB, Christensen J, Madsen KM, Ostergaard JR, Olsen J (2006). The Danish National Hospital Register is a valuable study base for epidemiologic research in febrile seizures. J Clin Epidemiol.

[31] Vigevano F, Kirkham FJ, Wilken B, Raspall-Chaure M, Grebla R, Lee D, Werner-Kiechle T, Lagae L (2018). Effect of rescue medication on seizure duration in non-institutionalized children with epilepsy. Eur J Paediatr Neurol.

[32] Visser AM, Jaddoe VW, Breteler MM, Hofman A, Moll HA, Arts WF (2012). Frequent fever episodes and the risk of febrile seizures: the Generation R study. Eur J Paediatr Neurol.

[33] Westin E, Sund Levander M (2018). Parent’s experiences of their children suffering febrile seizures. J Pediatr Nurs.

[34] Yilmaz D, Arhan E, Yuksel D, Ozçelik A, Senbil N, Serdaroglu A, Gurer YK (2008). Attitudes of parents and physicians toward febrile seizures. Clin Pediatr (Phila).

