# Supplementary material for: Febrile seizures: perceptions and knowledge of parents of affected and unaffected children
Source: Eur J Pediatr. 2021 Dec 7;181(4):1487–95. doi: 10.1007/s00431-021-04335-1 (PMC8648401; doi:10.1007/s00431-021-04335-1)
Supplement: Supplementary file 1 — Supplementary file1 (PDF 123 KB) [file 431_2021_4335_MOESM1_ESM.pdf]

Supplement 1
Febrile seizures: Perceptions and knowledge of parents of affected and unaffected children

Questionnaire

For parents of children with at least one febrile seizure (FS-group)

1.) a) Did you realize your child had a febrile seizure?
□ Yes
□ No
□ No statement

b) If no, what did you think happened? (open question)

2.) a) What was the dominating feeling when your child had a febrile seizure? (open question)

b) On a scale from 0 - 10 (0=least pronounced to 10=most pronounced), how strongly would you rate the feeling?

3.) Who told you, your child had a febrile seizure?
□ A physician
□ Someone else. Who? (open question)
□ No one.
□ I do not remember.

4.) Which issues were addressed in the first consultation about the diagnosis?

Acute measures to be taken in case of a febrile seizure
□ Yes □ No □ No statement
Long-term consequences of febrile seizures
□ Yes □ No □ No statement
Frequency of febrile seizures
□ Yes □ No □ No statement
Measures to prevent febrile seizures
□ Yes □ No □ No statement
Risk factors for febrile seizures
□ Yes □ No □ No statement
Acute complications of febrile seizures
□ Yes □ No □ No statement

5.) Changes after the febrile seizure

For parents of a child with at least one previous febrile seizure

a) Were there any general changes in the handling of your child?
□ Yes □ No □ No statement
b) Do you take your child's temperature more often?
□ Yes □ No □ No statement

c) Do you give your child medicine to lower his/her fever earlier?
□ Yes □ No □ No statement
d) At what temperature do you start giving your child medicine to lower his/her fever? (open question)

e) Do you see your pediatrician more often when your child has a fever?
□ Yes □ No □ No statement

For parents of a child with a first febrile seizure:

a) Will there be any general changes in the handling of your child?
□ Yes □ No □ No statement
b) Will you take your child's temperature more often?
□ Yes □ No □ No statement
c) Will you give your child medicine to lower his/her fever earlier?
□ Yes □ No □ No statement
d) At what temperature will you start giving your child medicine to lower his/her fever? (open question)

e) Will you see your pediatrician more often when your child has a fever?
□ Yes □ No □ No statement

For parents of children with at least one febrile seizure (FS-group) and parents of children who had not experienced a febrile seizure thus far (control group)

6.) For parents in the FS-group

Did you inform yourself on the topic of febrile seizures before your child was affected?
□ Yes □ No

For parents in the control group

Have you informed yourself on the topic of febrile seizures yet?
□ Yes □ No

7.) Which measures would you take in case of a febrile seizure?

Clear the surroundings
□ Yes □ No □ No statement

Calm the child
□ Yes □ No □ No statement

Administer an anti-seizure medication if available
□ Yes □ No □ No statement

Call the emergency
□ Yes □ No □ No statement

Document the length of the seizure
□ Yes □ No □ No statement

Administer fever medication
□ Yes □ No □ No statement

Put a solid object into the child's mouth
□ Yes □ No □ No statement

8.) Do you know about possible negative consequences resulting from a febrile seizure?

Injuries
□ Yes □ No □ No statement

Other febrile seizures
□ Yes □ No □ No statement

Suffocation of the child
□ Yes □ No □ No statement

Epilepsy
□ Yes □ No □ No statement

Developmental disorder
□ Yes □ No □ No statement

9.) a) Do you feel adequately prepared for a febrile seizure in your child?

□ Definitely
□ Rather yes
□ Partly
□ Rather no
□ Definitely no
□ I cannot judge my preparedness

b) What measures would you need to feel better prepared? (open question)

10.) Sociodemographic data parent

a) Gender ◻ Male ◻ Female

b) Age (years)

c) Education

◻ No school graduation
◻ "Hauptschule"/"Realschule" (schools that prepare mainly for vocational careers)
◻ "Abitur" (i.e. the diploma for entry into higher education)
◻ No statement

d) Profession (open question)

11.) Sociodemographic data child

a) Gender ◻ Male ◻ Female

b) Age (months)

c) Number of febrile seizures (n)

d) Fever associated with the current hospitalization?

◻ Yes ◻ No
